# Whole-Genome Sequence of *Listeria monocytogenes* Type Strain 53 XXIII

**DOI:** 10.1128/genomeA.00970-14

**Published:** 2014-09-25

**Authors:** K. W. Davenport, H. E. Daligault, T. D. Minogue, K. A. Bishop-Lilly, D. C. Bruce, P. S. Chain, S. R. Coyne, K. G. Frey, J. Jaissle, G. I. Koroleva, J. T. Ladner, P.-E. Li, G. F. Palacios, C. L. Redden, M. B. Scholz, H. Teshima, S. L. Johnson

**Affiliations:** a Los Alamos National Laboratory, Los Alamos, New Mexico, USA; b Diagnostic Systems Division (DSD), United States Army Medical Research Institute of Infectious Diseases (USAMRIID), Fort Detrick, Maryland, USA; c Naval Medical Research Center (NMRC)-Frederick, Fort Detrick, Maryland, USA; d Henry M. Jackson Foundation, Bethesda, Maryland, USA; e Center for Genome Sciences (CGS), USAMRIID, Fort Detrick, Maryland, USA

## Abstract

*Listeria monocytogenes* causes the food-borne illness listeriosis that primarily infects “vulnerable” groups (e.g., elderly adults, pregnant women, very young children, and the immunocompromised). We sequenced the genome of *L. monocytogenes* XXIII (ATCC 15313) and assembled it into a single scaffold (three contigs) and deposited the annotated assembly into GenBank as accession no. JOOX00000000.

## GENOME ANNOUNCEMENT

First isolated from rabbits in 1926, *Listeria monocytogenes* has been the causative agent of several food-borne outbreaks with a mortality rate of up to 30% (1, 2). *L. monocytogenes* XXIII (ATCC 15313) is the type strain and is commonly used in laboratory studies.

High-quality genomic DNA was extracted from a purified isolate using a QIAgen Genome Tip-500 at USAMRIID-Diagnostic Systems Division (DSD). Specifically, a 100-mL bacterial culture was grown to stationary phase and nucleic acid extracted as per manufacturer’s recommendations. A 100-bp Illumina library (300-fold coverage) was constructed and sequenced, as well as a separate long insert paired-end library (6,640- ± 1,660-bp insert, 16-fold coverage, 454 Titanium). The two libraries were assembled together in Newbler (Roche) and the consensus sequences were computationally shredded into 2-kbp overlapping fake reads (shreds). Raw reads were also assembled in Velvet and those consensus sequences were computationally shredded into 1.5-kbp overlapping shreds (3). Next, all draft data were assembled with Allpaths and the consensus sequences were computationally shredded into 10-kbp overlapping shreds (4). The consensus shreds from Newbler, Velvet, and Allpaths were then integrated with a subset of the long-insert read pairs using parallel Phrap. Possible misassemblies were corrected and some gap closure accomplished with manual editing in Consed (5–7).

Automatic annotation of the final (3 contig) assembly utilized an Ergatis based workflow at Los Alamos National Laboratory (LANL) with minor manual curation. The 2.85-Mbp (38% G+C content) assembly includes 2,781 coding sequences, 11 rRNA sequences, and 58 tRNA sequences. A preliminary analysis located multiple virulence factors (8), including *inlA,B,C,J*, *plcA, B*, and iap.

### Nucleotide sequence accession number.

This genome is available as accession no. JOOX00000000.
